# Comparative performance evaluation of bisulfite- and enzyme-based DNA conversion methods

**DOI:** 10.1186/s13148-025-01855-7

**Published:** 2025-04-03

**Authors:** Roy B. Simons, Faidra Karkala, Marta M. Kukk, Hieab H. H. Adams, Manfred Kayser, Athina Vidaki

**Affiliations:** 1 Department of Genetic Identification, Erasmus MC University Medical Center Rotterdam, Rotterdam, The Netherlands; 2 Department of Clinical Genetics, Erasmus MC, University Medical Center Rotterdam, Rotterdam, The Netherlands; 3 Present Address: Department of Clinical Genetics, Maastricht University Medical Center+, Maastricht, The Netherlands

**Keywords:** Bisulfite conversion, Enzymatic conversion, DNA methylation, Epigenetics, Method comparison

## Abstract

**Background:**

Bisulfite conversion (BC) has been the gold standard in DNA methylation profiling for decades. During this chemical process, non-methylated cytosines are converted into uracils, while methylated cytosines remain intact. Despite its popularity, BC has major drawbacks when used for sensitive applications with low-quality and -quantity DNA samples, such as the required large amount of DNA input, the caused DNA fragmentation and loss, and the resulting reduced sequence complexity. Lately, to account for BC-related disadvantages the first commercial enzymatic conversion (EC) kit was launched. While EC follows the same conversion principle as BC it uses two enzymatic steps instead of one chemical step with BC. In this study, we validated and compared the conversion performance of the most widely used BC and EC kits using a multiplex qPCR assay (qBiCo) we recently developed, which provides several indexes: conversion efficiency, converted DNA recovery and fragmentation.

**Results:**

Firstly, we implemented and standardized both DNA conversion methods. Secondly, using qBiCo, we performed a developmental validation for both conversion approaches, including testing the following parameters: repeatability, reproducibility, sensitivity and robustness. Regarding conversion efficiency, both methods performed similarly, with the limit of reproducible conversion being 5 ng and 10 ng for BC and EC, respectively. The recovery, however, is structurally overestimated for BC: 2.3 ± 0.7 and 0.7 ± 0.2 for EC. In contrast, degraded DNA input resulted in high fragmentation values after BC and low-medium values for EC (14.4 ± 1.2 and 3.3 ± 0.4, respectively). Finally, we converted 10 ng of 22 genomic DNA samples using both methods. We observed an overestimation of the BC DNA recovery (130%) and a low recovery for EC (40%).

**Conclusions:**

Our findings indicate that both DNA conversion methods have strengths and weaknesses. BC shows a high recovery, whereas EC does not cause extensive fragmentation that is characteristic to BC. EC is, therefore, more robust to the analysis of degraded DNA such as forensic-type or cell-free DNA, at least for the genomic DNA inputs tested here. We believe that the low recovery of EC could be improved by further optimizing and automating the bead-based cleanup steps. Overall, our study provides the first independent benchmarking of bisulfite- and enzyme-based conversion kits.

**Supplementary Information:**

The online version contains supplementary material available at 10.1186/s13148-025-01855-7.

## Background

DNA methylation is among the most studied human epigenetic markers and is associated to a wide range of relevant biological processes such as aging and disease. CpG dinucleotides (or CpG sites) are concentrated in CpG islands often located at gene promoter sites. Cytosine methylation at CpG sites is involved in gene activity regulation, generally resulting in silencing of gene expression [1]. Due to its important role in transcription regulation, DNA methylation biomarkers can be a valuable source of information for accurate inference of biological age and other common phenotypes and for early detection or progression of disease [2–5]. Moreover, DNA methylation markers are used in applied fields such as in forensics to predict age and habits that involve environmental interaction such as smoking [6–9].

To measure CpG methylation, DNA usually needs to be converted to create sequence-based differences. Bisulfite conversion (BC) has been the gold standard for analysis of CpG methylation for decades. During this process, incubation of DNA with sodium bisulfite results in the conversion of unmethylated cytosine to uracil, while methylated cytosines remain intact. Subsequently, analyzing the ratio of converted to non-converted cytosines through, e.g., DNA sequencing, provides the methylation status of CpG sites [10–14]. Particularly when coupled with whole-genome sequencing or methylation microarrays, BC has extensively been used to discover novel biomarkers toward the development of diagnostic tests [15, 16].

Bisulfite conversion, however, has at least three major drawbacks. Firstly, incomplete conversion or preferential degradation of unmethylated DNA can influence measurement by overestimation of methylation levels [17]. While current bisulfite conversion kits have been available for decades and are considered robust, some often fail to convert efficiently, or at least in low amounts (less than 10 ng input DNA), and the possibility of random failure due to experimental issues cannot be excluded either [18]. Also, we acknowledge that CpG methylation is widely underrepresented genome-wide compared to non-CG context, hence in theory it could be affected less likely. However, in targeted assay investigations primers are often specifically designed to bind to non-CG sequences to distinguish non-converted DNA. Therefore, incomplete non-CG conversion can still indirectly impact methylation quantification. This is highly relevant as CpG methylation lately finds its way into the clinic via translating findings into targeted diagnostic tests for phenotype correlation or prediction, such as for the estimation of biological aging rate, or the diagnosis of cardiovascular diseases, colorectal cancer liver metastasis and many more diseases [19–24]. Secondly, the harsh chemical treatment with sodium bisulfite causes severe DNA fragmentation, thereby limiting further downstream analysis and resulting in potentially substantial loss of the converted DNA template [25]. Thirdly, the complexity of the genome as a whole decreases as all non-CpG cytosines that are expected to be unmethylated are converted to uracils. During polymerase chain reaction (PCR) with a U-tolerating polymerase [26], the uracils are converted to thymines, leaving a converted DNA sequence with skewed base composition. This T-rich sequence substantially decreases the sequence complexity from a 4- to a 3-letter genome, which further decreases the specificity of primer or probe binding in subsequent PCR or capture experiments, respectively.

**Fig. 1:**
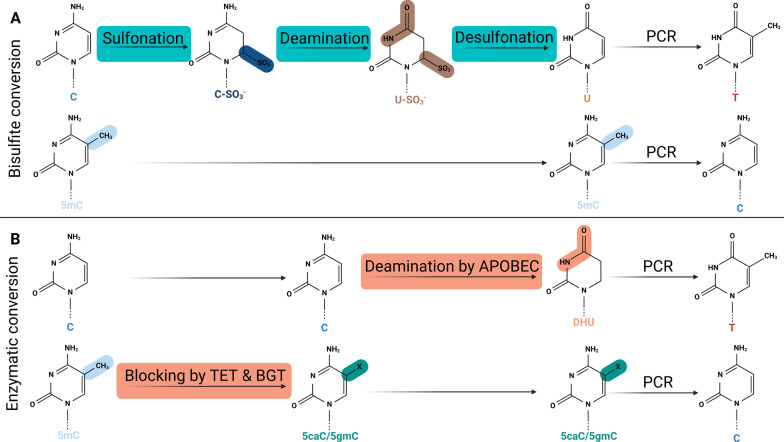
Schematic overview of two DNA conversion approaches for CpG methylation detection. **A** Bisulfite conversion, **B** enzymatic conversion. X denotes either $\hbox {CH}_{2}$O or C${_7}\hbox {H}_{13}\hbox {O}_{6}$ for 5-carboxyl cytosine (5caC) and 5-(beta-glucosyloxymethyl) cytosine (5gmC), respectively. Enzymatic steps are shown in orange boxes, whereas chemical steps are shown in blue boxes. Note that EC makes use of a blocking step followed by a conversion step, whereas BC uses consecutive conversion steps. Both methods result in an unmethylated cytosine-to-thymine conversion. Created with BioRender.com

To overcome the first two of these challenges, a non-chemical-based approach, enzymatic conversion (EC), has been developed [27–30]. EC solely involves enzymatic steps as opposed to the chemical conversion of BC, promising a more gentle DNA treatment (Fig. 1). First, methylated cytosines (5mC) are blocked from being converted with a carboxyl group by TET oxidation and glucose by T4-BGT glycosylation, followed by deamination of the unmethylated cytosines into dihydrouracil (DHU) by APOBEC. During PCR, DHU is replaced by thymine, resulting in a similar unmethylated cytosine-to-thymine conversion as by BC, but without the use of the harsh chemical conditions [31]. Due to the enzymatic nature of EC, a lower amount of fragmentation is expected, which is beneficial especially for low-quality samples such as cell-free or forensic-type DNA [27, 29, 32].

Both BC and EC comprise of standard molecular biology laboratory steps used when handling DNA. There is a high diversity in the currently available commercial BC kits, whereas at the moment there is only one commercially available EC kit. For instance, considering one of the most popular BC kits (EZ DNA methylation kit from Zymo Research), BC has a longer protocol time due to a 16 h incubation step with a column-based purification. On the other hand, EC only has a total of 4.5 h incubation steps, but two tedious and time-consuming bead-cleanup steps, when performed manually. DNA input for BC ranges from 500 pg to 2 $\upmu$g, whereas for EC spans a narrower range of 10–200 ng (Table 1).

**Table 1:** Overview and comparison of genomic DNA conversion protocols included in this study

Characteristics	Bisulfite conversion	Enzymatic conversion
DNA input range	0.5–2000 ng	10–200 ng
Protocol incubation time	12–16 h	6 h
Cost per conversion	€2.91	€6.41
Elution volume range	$\ge$ 10 $\upmu$l	20 $\upmu$l

Recently, to overcome the current practice of blindly trusting the performance of BC, particularly when using low DNA amounts, we developed a multiplex TaqMan-based quantitative PCR method (qBiCo) that can be used as a quality control step prior to conducting costly DNA methylation analysis such as sequencing or microarray experiments. In our recent study, we showcased that qBiCo can reproducibly and sensitively assess converted DNA samples on various qPCR instruments in terms of conversion efficiency, recovery and fragmentation, based on the detection of both single-copy genes and repetitive elements [18]. Having developed qBiCo puts us in an advantaged position to compare and validate the performance of various conversion kits regardless of conversion approach and manufacturer.

In this study, we aimed to provide the scientific community with a first systematic evaluation of the current commercially available BC and EC gold standards based on qBiCo. First, we adopted a workable and comparable protocol for both conversion methods. Second, we performed a developmental validation for each kit, taking into account various parameters such as reproducibility, robustness in terms of input DNA quality and protocol variations. Finally, we compared the performance of both kits using a small amount (10 ng) of the same set of blood-derived DNA samples to give an insight into the conversion performances on low-quality and -quantity samples.

## Results

### Choice of DNA conversion and qPCR assessment approaches

For BC, the EZ DNA methylation kit (Zymo Research) was chosen as the representative kit as it currently is one of the most popular bisulfite conversion kits judged from the literature as well as recommended by the company itself for use with Illumina Infinium MethylationEPIC BeadChip array that is the gold standard in methylation discovery studies. Additionally, it was recently ranked as the highest-performing kit among more than ten commercial BC kits in our previous investigation [18]. For EC, the NEBNext^®^ Enzymatic Methyl-seq Conversion Module (New England Biolabs) was chosen as the only commercially available kit of its kind thus far. Given that both methods have different requirements and include diverse experimental steps, we slightly adjusted their protocols to allow for a fair comparison (Table 1). Particularly, for EC, and after consultation with the manufacturer to test a beta protocol more targeted for difficult, low input and already degraded DNA samples, no fragmentation prior to conversion was performed (see Methods for details). Importantly, that way we ensure that we solely measure the effect of the conversion method on the observed fragmentation levels. For BC, the elution volume was increased to 20 $\upmu$l to match the EC protocol.

To evaluate the two DNA conversion approaches, we employed the qPCR-based tool qBiCo (v2) that we have recently developed [18]. In brief, qBiCo is a 5-plex qPCR assay targeting a set of single-copy and repetitive sequences of the human DNA in order to assess several BC performance parameters: genome-wide conversion efficiency, converted DNA concentration and converted DNA fragmentation. First, to calculate global conversion efficiency, we employ two assays (Genomic/Converted) targeting the genomic and converted version of the multi-copy human L1 repetitive element (LINE-1) ($\sim$200 copies across the genome). Second, to calculate converted DNA concentration, we employ an assay (Short) targeting the converted version of the single-copy *hTERT* gene, previously used in commercial genomic DNA quantification assays. Third, to calculate converted DNA fragmentation, we additionally employ an assay (Long) targeting the converted but longer version of another single-copy *TPT1* gene and compare it with the observed copies of the Short assay. More details on the methodology can be found in the Methods section. In our previous efforts, qBiCo was thoroughly optimized, validated and tested by using different bisulfite conversion kits, genomic DNA inputs and samples of varying quality [18], which also guided our experiments here.

Here, the performance of each separate qBiCo assay was based on PCR efficiency and linear fit of the standard curves. The lowest obtained *R*^2^ value for an assay was 0.95 with a mean value of 0.99 ± 0.01. The mean PCR efficiency was 90.3 ± 9.8% (Table 2). All qBiCo standard curves and performance parameters for each assay can be found in Supplementary file 1.

**Table 2:** Average performance of individual qBiCo assays

Assay	PCR efficiency (%)	*R*^2^-value
Genomic	95 ± 9	0.989 ± 0.009
Long	87 ± 11	0.994 ± 0.008
Short	88 ± 10	0.991 ± 0.012
Converted	91 ± 9	0.996 ± 0.005

### Evaluation of global conversion efficiency

**Fig. 2:**
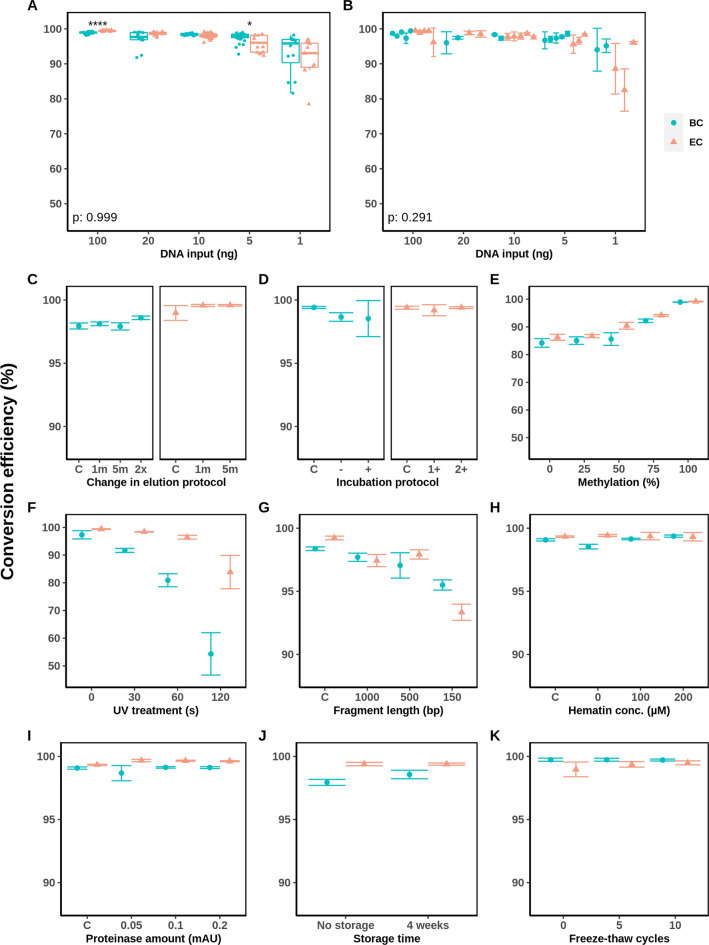
Developmental validation of BC and EC DNA conversion methods in terms of conversion efficiency based on the recently developed qPCR QC tool qBiCo [18]. Several parameters were assessed including: **A** repeatability for intra-experimental variation; **B** reproducibility and sensitivity for inter-experimental variation; effects of **C** elution and **D** incubation time in the conversion methods protocol; **E** artificial methylation of commercial gDNA standards; robustness in terms of **F** UV treatment and **G** sonication prior to conversion; inhibition by **H** hematin and **I** proteinase; stability of converted gDNA by **J** storage time and **K** freeze-thaw cycles. The genomic DNA input for all conditions (**C**–**K**) was 100 ng

Our study goal was to compare the global sample conversion efficiencies of both BC and EC methods when the same DNA samples were treated under different experimental conditions (Fig. 2). We considered conversion efficiency the most important parameter as it can determine whether a sample should be considered for downstream analysis, or not. qBiCo provides the conversion efficiency as one outcome parameter, which is estimated by comparing the detection of two fluorescently labeled fragments: the converted and non-converted (genomic) version of a DNA sequence within the human L1 repetitive element, found hundreds of times across the genome [18].

Firstly, we tested the effect of the initial DNA input on the conversion efficiency and how repeatable/reproducible our measurement is. When comparing a single BC and EC conversion experiment, no significant difference in conversion efficiency was detected with the DNA input as covariate (p = 0.999). However, at 100 ng, the conversion efficiencies were significantly different, namely, 98.9 ± 0.3% and 99.4 ± 0.2% for BC and EC, respectively. (Fig. 2A: ****: *p* < 0.0001), probably driven by the very low variance at this high amount. Using 100 ng of input DNA which falls within the optimal range for both methods, the conversion efficiency was 98.5 ± 1.0% (BC) and 98.7 ± 2.1% (EC), respectively (Fig. 2B). Subsequently, when decreasing the DNA input five (20 ng) and ten times (10 ng), but still within the suggested limits from the manufacturers for both methods, the conversion was detected as 96.7 ± 2.3% (10 ng: 97.8 ± 0.7%) and 98.7 ± 0.7% (10 ng: 97.9 ± 0.9%), respectively (Fig. 2B). Interestingly, when further reducing the input and treating sub-optimal DNA amounts (5 and 1 ng), the conversion efficiency decreased to 97.5 ± 1.4% (1 ng: 94.6 ± 4.4%) and 96.6 ± 1.8% (1 ng: 87.2 ± 7.6%) for BC and EC, respectively (Fig. 2B). For all amounts, no statistically significant difference between the two conversion methods was observed while including the DNA input as a covariate (*p* = 0.291), although we critically acknowledge the small sample size (*n* = 3). Per DNA input amount, no statistically significant differences in conversion efficiency were detectable when taking into account the inter-experimental variation between the conversion methods. Additionally, an increasing variance was detected at DNA input amounts of 1 ng, indicating that conversion was not reproducible at this level op input DNA.

Secondly, we tested the effect of various other experimental conditions on the conversion efficiency, in terms of either protocol variations or sample characteristics. The effect of the changes to the conversion method protocols were not statistically significant, resulting in conversion efficiencies of 98.2 ± 0.3% and 99.4 ± 0.4% for the different elution protocols and 98.9 ± 0.7% and 99.3 ± 0.3% at the various incubation protocols for BC and EC, respectively (Fig. 2 C, D). Overall, the conversion efficiency decreased with a decreasing methylation status when treating artificially methylated DNA going down. Especially at a methylation status of 50% or lower, the conversion efficiency decreased to 85.0 ± 1.7% and 87.8 ± 2.2% for BC and EC, respectively (Fig. 2E). This effect was measured earlier when using qBiCo [18]. Both robustness tests showed a decrease in conversion efficiency after either UV treatment or sonication of the gDNA prior to conversion. Namely, at a fragment length of 1000 or 500 bp, the conversion efficiencies had a minor decrease (BC: 97.4 ± 0.7%;EC: 97.6 ± 0.4%), whereas at a median fragment length of 150 bp, these values lowered to 95.5 ± 0.4% and 93.3 ± 0.6% for BC and EC, respectively (Fig. 2F). BC was significantly more prone to the effect of pre-UV-treated DNA. At 120 s of UV treatment of the input DNA, conversion efficiencies decreased to 54.3 ± 7.7% for BC and 83.9 ± 6.0% for EC (Fig. 2G). Both common contaminants of gDNA, hematin and proteinase, and both storage conditions did not affect the conversion efficiency (Fig. 2 H–K). No statistical evaluations were made for these parameters due to the small set of data points.

### Evaluation of converted DNA recovery

**Fig. 3:**
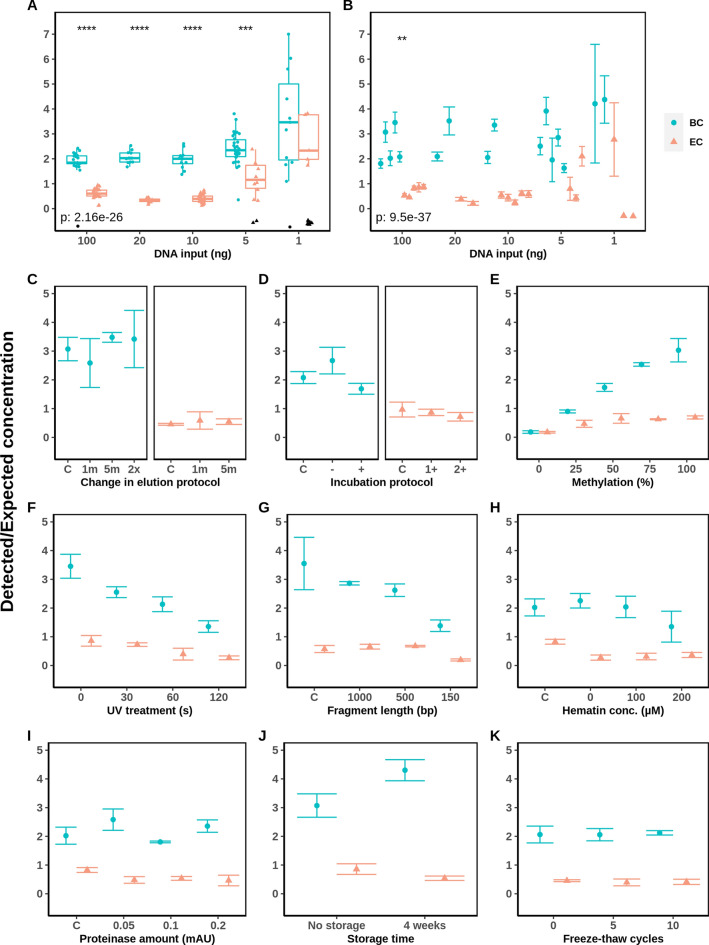
Developmental validation of BC and EC DNA conversion methods in terms of converted DNA recovery based on the recently developed qPCR QC tool qBiCo [18]. Several parameters were assessed including: **A** repeatability for intra-experimental variation; **B** reproducibility and sensitivity for inter-experimental variation; effects of **C** elution and **D** incubation time in the conversion methods protocol; **E** artificial methylation of commercial gDNA standards; robustness in terms of **F** UV treatment and **G** sonication prior to conversion; inhibition by **H** hematin and **I** proteinase; stability of converted gDNA by **J** storage time and **K** freeze-thaw cycles. The genomic DNA input for all conditions (**C**–**K**) was 100 ng. Missing values for **A** are displayed at y = 0 in black

In order to compare the recovery of converted DNA between both BC and EC methods, the same DNA samples were treated under different experimental conditions (Fig. 3). The amount of recovered converted DNA was determined by quantification of a single-locus converted DNA sequence (*hTERT* gene) in the samples after conversion by the qPCR assay qBiCo [18]. This ensures enough DNA is recovered for follow-up analyses, such as for whole-genome or targeted bisulfite sequencing. We calculated converted DNA recovery dividing the amount of recovered converted DNA by the initial DNA input.

Firstly, the repeatability/reproducibility of the recovery was compared for both conversion methods. Generally, the recovery index for BC was significantly higher than for EC (Fig. 3) (*p* = 2.16e$-$26). When compared in a single experiment, BC shows recoveries of 1.9 ± 0.2, 2.1 ± 0.2 and 2.0 ± 0.4 for 100, 20 and 10 ng of input DNA, respectively. In contrast, for EC these values were 0.6 ± 0.2, 0.4 ± 0.1 and 0.4 ± 0.2 (Fig. 3A). The recovery was overestimated for all BC samples shown by their values exceeding 1. At the sub-optimal DNA input amounts of 5 and 1 ng, the recoveries were systematically overestimated when above the limit of detection, showing values of 2.4 ± 0.6 (1ng: 3.6 ± 1.9) and 1.2 ± 0.7 (1ng: 2.7 ± 1.0) for BC and EC, respectively (Fig. 3A). Additionally, the number of missing values increased greatly at these low amounts of input DNA. When taking into account inter-experimental differences at the representative DNA input amount of 100 ng, the recoveries were 2.5 ± 0.7 and 0.7 ± 0.2 for BC and EC, respectively (Fig. 3B), showing to be significantly different between BC and EC with the DNA input as a covariate (*p* = 9.5e$-$37). Also, when reaching the limit of detection of this index at 1 ng input DNA, the recovery is overestimated, as shown by the increase in recovery from 2.5 to 4.3 for BC and 0.7 to 2.7 for EC (Fig. 3B).

Secondly, no clear effects were found of the changes in elution strategy and incubation time in the conversion protocols (Fig. 3 C,D). Interestingly, low artificially methylated DNA mixtures were harder to recover after conversion by both BC and EC. The decrease in recovery from 100% methylated DNA down to 0% methylated DNA appeared stronger after BC than EC, with decreasing linearly from 3.0 ± 0.4 and 0.7 ± 0.1 to 0.18 ± 0.04 and 0.17 ± 0.03 for BC and EC, respectively (Fig. 3E). EC showed to be more robust for gDNA samples that underwent UV treatment or sonication prior to conversion, shown by a lower relative decrease of recovery. After conversion of UV-treated samples, the recovery decreased 2.5-fold for BC (3.5 ± 0.4 to 1.4 ± 0.2) and 3-fold for EC (0.9 ± 0.2 to 0.3 ± 0.1) at 120 s of UV treatment. A similar decrease from 3.6 ± 0.9 to 1.4 ± 0.2 for BC and 0.6 ± 0.1 to 0.2 ± 0.04 for EC was seen at sonication to a 150 bp fragments (Fig. 3 F,G). The addition of the solute, NaOH, in which hematin was dissolved, decreased the recovery of the EC DNA samples from 0.8 ± 0.1 to 0.3 ± 0.1, but no decrease was seen for BC: 2.0 ± 0.3 to 2.3 ± 0.3 (Fig. 3H). Afterward, no further decrease in recovery was detected when adding hematin. Both common contaminants of gDNA, hematin and proteinase, and both storage conditions did not affect the recovery in BC and EC (Fig. 3 I–K).

### Evaluation of converted DNA fragmentation

To determine the effect of both conversion methods on the fragmentation of input DNA, we measured the fragmentation level of the converted DNA in various experimental conditions. This level of fragmentation was determined by the quantification of a short (85 bp) and a long (235 bp) fragment, targeting the *hTERT* and *TPT1* genes in the samples after conversion by the qPCR assay qBiCo [18]. This follows the same principle as in current commercially available quantification kits for genomic DNA with an incorporated degradation level indication, such as the Quantifiler Trio (Thermo Fisher Scientific) or PowerQuant (Promega) kits; however, now targeting the converted DNA sequence. It is important to emphasize here that the fragmentation level is a qualitative index, showing degradation of the DNA sample after conversion.

**Fig. 4:**
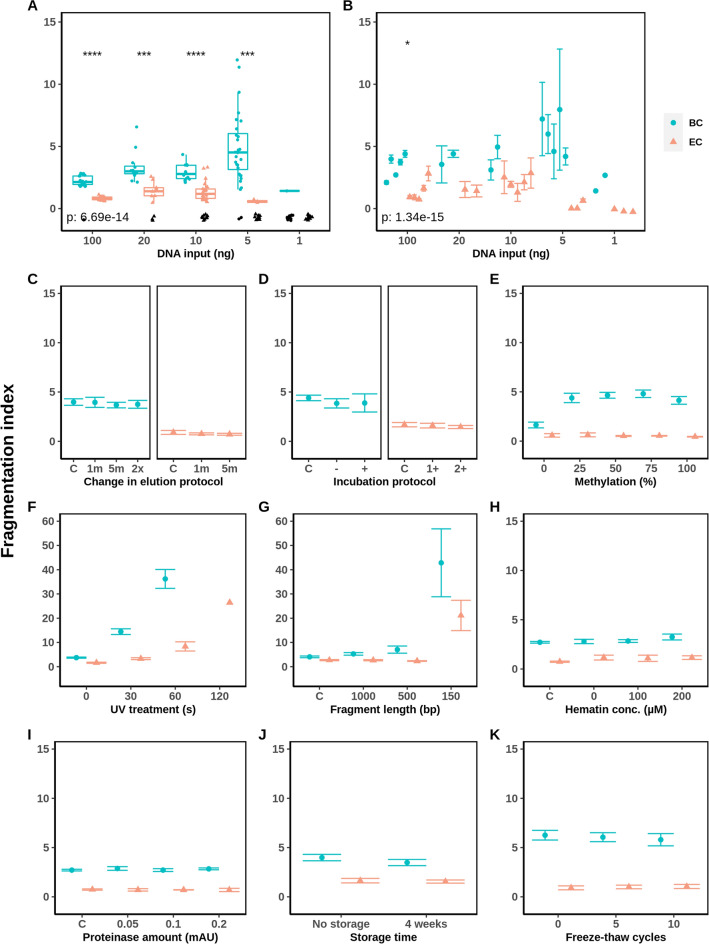
Developmental validation of BC and EC DNA conversion methods in terms of fragmentation index based on the recently developed qPCR QC tool qBiCo [18]. Several parameters were assessed including: **A** repeatability for intra-experimental variation; **B** reproducibility and sensitivity for inter-experimental variation; effects of **C** elution and **D** incubation time in the conversion methods protocol; **E** artificial methylation of commercial gDNA standards; robustness in terms of **F** UV treatment and **G** sonication prior to conversion; inhibition by **H** hematin and **I** proteinase; stability of converted gDNA by **J** storage time and **K** freeze-thaw cycles. The genomic DNA input for all conditions (**C**–**K**) was 100 ng. Missing values for **A** are displayed at y = 0 in black

Firstly, the fragmentation indices for both conversion methods were compared (Fig. 4). In a comparison of both conversion methods performed in a single experiment, BC shows a 2.7-fold higher fragmentation index than EC with values of 2.2 ± 0.4 and 0.8 ± 0.1, respectively (Fig. 4A; *p* = 3.96e$-$14). When reproduced over various experiments, this significant difference between the two methods was holding (p = 5.81e$-$15), with a value of 1.4 ± 0.8 for EC and 3.4 ± 0.9 for BC at the reference amount of 100 ng gDNA (Fig. 4B). Generally, BC fragments the DNA to a higher degree than EC, as expected due to the harshness of sodium bisulfite [25]. The detected fragmentation index was stable for both methods over 100, 20 and 10 ng of input DNA. At 5 ng of input DNA for BC, however, the fragmentation index increased from 0.9$-$1.3 to 3.0, but was under the limit of detection for EC. A point to note here is that the amplified large fragments of the *hTERT* gene are harder to detect than the other qBiCo assays due to its increased length, often resulting in missing values at lower converted DNA inputs as also seen here (Fig. 4A).

Secondly, two parameters which are expected to influence the fragmentation index are UV treatment and sonication prior to conversion, showing the importance of the quality of the initial gDNA samples. EC was more robust to both of these treatments as the fragmentation index for the BC samples was higher for all conditions. After 60 s, a 5-fold increase in the fragmentation index happened at EC from 1.6 ± 0.2 to 8.4 ± 1.8, whereas a 10-fold increase was measured at BC from 3.8 ± 0.2 to 36.2 ± 3.9 (Fig. 4F). These values showed an exponential increase of the fragmentation index in case of UV treatment prior to conversion, indicating that already damaged DNA fragments faster. A similar increase in fragmentation index was seen at the sheared DNA of 150 bp fragment length. Here, compared to the non-sheared samples, an 11-fold increase was seen at BC from 4.0 ± 0.3 to 42.8 ± 14.0 and an 8-fold increase at EC from 2.7 ± 0.2 to 21.1 ± 6.3 (Fig. 4G). Additionally, at 0% methylated standards the fragmentation index was detected at a 2.7-fold decreased fragmentation level for the BC samples at 1.6 ± 0.3 as opposed to the 4.5 ± 0.4 index for the other methylation levels. None of the other parameters seemed to have an effect on the fragmentation index for both conversion methods.

### Overall DNA conversion method performance based on qBiCo

While comparing individual conversion performance-related indexes can give us valuable insights into each method’s strengths and weaknesses, we also aimed to holistically evaluate each conversion method and how the measurement of each index could influence one another.

According to the manufacturers of conversion kits, global conversion efficiency should be > 99% However, no minimum threshold has been decided among the scientific community as it has not been possible so far to measure conversion efficiency prior to DNA sequencing. While we were able to detect converted DNA when lower amounts are converted, combining the results of all three indices showed that the reproducible limit of detection for both BC and EC was 10 ng. At this amount, which corresponds to 0.5 ng of converted DNA input for each of the two technical replicates into qBiCo, we could confidently obtain measurements without statistically significant differences from the optical amount (100 ng). Not only was the variation of our measurements between replicates high, but we also obtained missing values. We believe this variation is caused by the conversion itself, rather that the qPCR detection, as we have previously shown that qBiCo can deliver reliable measurements down to 310 pg [18].

Next, as the fragmentation level increased, the reported conversion efficiency decreased, which was most evidently seen at samples treated with UV. It has been reported previously that for highly converted DNA samples the conversion efficiency cannot be accurately reported by qBiCo [18]. Additionally, both BC and EC seemed to exhibit a lower performance at artificially induced global (genome-wide) methylation percentages of 0–25%. Especially, the conversion efficiency decreased from 98–99% to 85–88%, while the recovery decreased to 0.14$-$0.22 for both conversion methods. In other words, with more degraded and less available DNA template, the detected conversion efficiency is less or less accurate. We also suspect these observations to be due to preferential degradation of unmethylated DNA [17]. Yet, it is important to reflect that very low methylated samples at the genome-wide scale are not encountered naturally, and are used here only for testing the limits of the technology.

### Application and variability at low-level DNA conversion (10 ng)

**Fig. 5:**
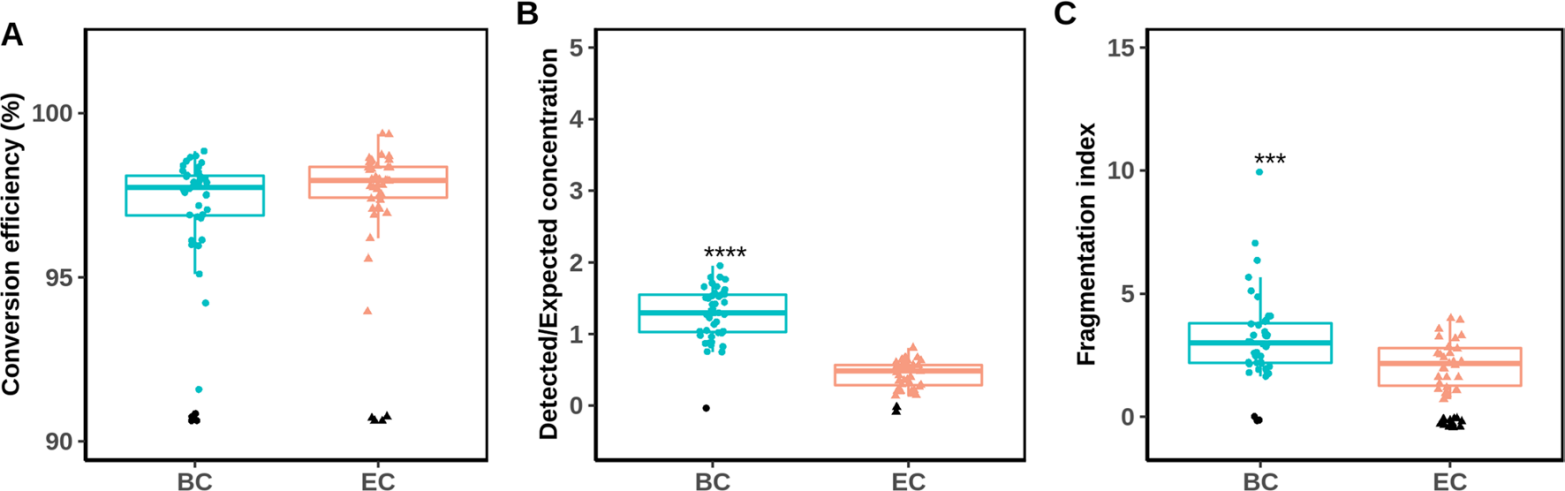
Comparison between BC and EC conversion performance using 22 whole blood gDNA samples (10 ng). Indices obtained by qBiCo [18] are shown on the *y*-axis: **A** conversion efficiency, **B** converted DNA recovery and **C** converted DNA fragmentation. Missing values are displayed at the bottom of the graphs in black

Finally, we aimed to apply both conversion methods to real-life settings and compared their performance side-by-side when the minimum DNA amount was converted (10 ng), which is often the case when dealing with difficult templates like forensic-type or cell-free DNA. We decided to employ the same experimental conditions as in the previous conversion performance validation experiments, as no large differences in performance were observed when changing the various experimental parameters, as presented above. To achieve this, we converted a small set of whole blood samples under the same experimental conditions that allowed us to further assess the variability and reproducibility of BC and EC on a larger scale. For this experiment, we converted 22 gDNA samples, whose qBiCo performance indices resulted in a similar trend as previously seen during validation (Fig. 5.) It is important to mention that to circumvent possible qBiCo batch effects, BC and EC samples were randomly mixed over two qBiCo assays. Sample conversion efficiencies were detected at 97.3 ± 1.4% for BC and 97.8 ± 1.0% for EC (Fig. 5A), matching the previous measurements during validation (97.8 ± 0.7% for BC and 97.9 ± 0.9% for EC) (Fig. 2B). Once again, a clear overestimation of the converted DNA recovery index was detected for bisulfite-converted samples with a mean recovery of 1.3 ± 0.3, which indicates systematic bias, while similarly, the converted DNA recovery for enzymatic converted samples was again low with a mean recovery of 0.4 ± 0.2 (Fig. 5B). Lastly, the mean fragmentation levels were measured at 3.3 ± 1.7 and 2.1 ± 0.9, for BC and EC, respectively (Fig. 5C). For various samples, one of the technical qPCR replicates resulted in an index below the limit of detection of qBiCo as seen previously at the DNA input amount of 10 ng. More specifically, there was one (5%) EC sample for which it was not possible to determine the conversion efficiency. Additionally, for 7 (32%) of the qBiCo measurements, the fragmentation indices were below the limit of detection. Only for one other sample, the fragmentation index could not be determined, due to missing data of both technical replicates.

## Discussion

Currently, there is a strong development in the field of DNA methylation analysis, with novel methods being proposed regularly based on short-read sequencing such as 5-letter-seq [33], Methyl-SNP-seq [34] and RIMS-seq (Rapid identification of methylase specificity) [35], which all make use of either bisulfite or enzymatic conversion (BC and EC). As DNA methylation analysis relies on successful DNA conversion, it is important to be able to fully evaluate the performance of this process as part of commercially available kits. Hence, the goal of our study was to systematically assess the performance of BC and EC and provide insights into the strengths and weaknesses of both conversion approaches. To the best of our knowledge, this is the first benchmarking and validation study of bisulfite and enzymatic conversion by a qPCR-based quality control assay, which in this case is qBiCo, recently developed by our group [18].

Overall, our findings indicate that both DNA conversion methods have their own strengths and weaknesses in terms of conversion efficiency, converted DNA recovery and fragmentation. On the one hand, EC shows to be more robust causing less degradation to the input genomic DNA, without affecting the conversion efficiency and fragmentation index as much as BC. BC, on the other hand, shows a higher converted DNA recovery ability, nevertheless, BC samples show an increased DNA fragmentation, which is known to be a side effect since its discovery [10, 12]. It is important to highlight here that all comparisons made in this study are limited to the BC and EC kits employed and should be considered carefully without making general statements on the approaches. qBiCo will enable the assessment of additional kits in future studies, so that each laboratory is able to identify the best performing kit depending on the sample type and application in question.

First, we evaluated the performance of BC and EC on their global conversion efficiency, which we were able to achieve for the first time by targeting a region of a repetitive element (LINE-1). Altogether, both conversion methods showed comparable conversion efficiencies over all parameters tested, except for UV treatment. UV treatment of gDNA prior to conversion, in combination with the additional fragmentation and DNA damage that occurs during BC, results in decreased conversion efficiency. This decrease in conversion efficiency at longer UV treatment time could be explained by the accompanied fragmentation index. At high fragmentation levels (Fragmentation index above 8 [18]), the conversion efficiency index was affected by a decreased fragment length, which is likely the result of the difference in target lengths of the qBiCo assays (converted: 148 bp, genomic: 119 bp). As the converted assay target is longer than the genomic assay target, the converted assay was affected by fragmentation more substantially than the genomic assay, resulting in a decrease in detected conversion efficiency. This emphasizes the importance of regarding all three indices together when assessing the conversion's performance of a sample. In comparison to the conversion efficiency index, the recovery index was not as highly affected by DNA damage by UV or DNA degradation by sonication as the target length of the qBiCo short assay is only 85 bp.

Secondly, we evaluated the performance of both conversion methods regarding their converted DNA recovery. For both methods, the recovery became increasingly complex with lower amounts of input DNA. The limit of detection was 5 ng for BC and 10 ng for EC. At lower amounts, the qBiCo assay resulted in missing values, as concentrations fall below its detection limit. However, as we previously showed, the limit of detection by qBiCo is 150 pg [18], which indicates a rather full loss of the DNA sample. Especially, the fragmentation index is also not obtainable at low input amounts. Also, the rather low converted DNA recovery by EC (on average 5 times lower than BC at 10 ng) could be caused by performing the bead-cleanup steps manually; particularly, due to the high viscosity of the solution after the APOBEC incubation as mentioned by the NEBNext^®^ Enzymatic Methyl-seq Conversion Module protocol (New England Biolabs). It would be interesting to study the effect of lowering the viscosity by diluting the sample prior to the bead cleanup, although that was not in the scope of this study. Other previously investigated methods to improve nucleic acid recovery when using magnetic beads include automation and the use of microfluidic devices [36, 37].

Additionally, it has been previously reported that the recovery of both the magnetic bead and column purification steps can be increased by using carrier DNA [38], thereby decreasing the limit of detection for both methods. Especially at the lower input amounts of 10, 5 and 1 ng, carrier DNA might help to recover the expected minute amounts of converted DNA.

Unexpectedly, it seems that the total amount of recovered converted DNA is overestimated, particularly for the BC-treated samples. In this study, we determined the converted DNA recovery by dividing the amount of recovered DNA by the amount of input DNA. For this calculation, we used data from two different quantification (qPCR) experiments - 1) for genomic DNA quantification (Quantifiler) prior to conversion and 2) for converted DNA quantification (qBiCo) post-conversion. As we know that each qPCR can have its own amplification efficiency depending on targets, amplicon lengths and PCR conditions, it is possible that the values are biased and hence, to obtain values of over 100% recovery. To address this issue of overestimation of the DNA recovery after conversion, as a future adjustment a spike-in synthetic DNA molecule could be used, which can be mixed with the gDNA samples prior to conversion. When assessing the conversion through a qPCR QC-assay, the spike-in can be added to the qPCR run for normalization of the recovery index.

Additionally, qBiCo has been optimized for the analysis of converted DNA, therefore, the recovery and fragmentation indices are not as trustworthy in presence of a sample with a conversion efficiency lower than 90% (e.g. 85% at the 0% methylated artificial DNA standards). As both the DNA concentration and fragmentation assays target converted DNA, these values are underestimated at decreased conversion efficiency, due to a decrease in qPCR efficiency at longer, not fully converted qPCR targets.

Thirdly, both methods exhibit strongly differing fragmentation indices. EC-converted DNA samples exhibit significantly lower fragmentation indices than BC-converted DNA samples, which is reported as the main advantage of EC by their inventors [31]. UV treatment prior to conversion seems to have a bigger effect on the performance of BC, whereas sonication is the same for BC and EC. This could be ascribed to the particular fragmentation that occurs by the harsh chemical process during BC. Interestingly, a longer incubation time, however, did not result in a measurable increase in fragmentation. Nevertheless, the combination of pre-conversion UV damage and fragmentation during BC itself can increase the fragmentation rate of the DNA during incubation with sodium bisulfite, as the detected fragmentation level increases exponentially. These conclusions can at least be drawn when using optimal DNA amount (100 ng); nevertheless, we do not expect that the level of degradation caused by both BC and EC would change drastically when lower DNA amounts are treated. On the other hand, if the sample is of too low quantity, degradation caused by conversion could substantially reduce the available template for downstream analysis, including for QC assessment by qBiCo. Future studies should be performed to test the combined impact of low-quality/quantity DNA samples in the performance of DNA conversion.

When already highly-fragmented sample needs to be analyzed, it is recommended to choose EC over BC to limit further fragmentation of the DNA due to the constant higher fragmentation rate observed for BC in this study. However, due to the lower recovery of EC, BC still remains the overall gold standard for low-template samples. These conclusions are in agreement with previously reported results, as Hong et al. [39] reported a converted DNA recovery with EC of half the amount obtained with BC [39]. Additionally, and more recently, when converting ancient DNA bisulfite treatment also showed higher yield of converted DNA compared to enzymatic conversion [40]. Next to the performance of both methods, the current price difference could also be a reason to continue using BC as at the time of our study, the EC kit was 2.2 times more expensive than the BC kit used.

As the EC kit was developed for sheared DNA, a reason for the lower recovery of EC might be due to a decreased elution of larger DNA fragments at the magnetic bead purification. To only measure the effects of the conversion itself the DNA shearing as a pre-processing step which could decrease the DNA input quality and quantity upfront was removed.

Additionally, various studies have compared the performance of multiple BC kits. On one hand, qPCR-based approaches investigated similar performance parameters as qBiCo although combined from separate reactions. Holmes et al. performed a triplex assay to simultaneously measure the converted and unconverted DNA amounts, thus determining the conversion efficiency and converted DNA recovery. To determine the DNA degradation samples were analyzed on an agarose gel [41]. Kint et al. used digital PCR with assays of increasing length to analyze BC-induced degradation [42]. On the other hand, performance analysis by sequencing-converted DNA samples led to accurate conversion efficiency statistics, not only on 5mC [43] but also on it oxidative derivatives, 5hmC, 5fC and 5caC [44]. Whereas, the sequencing-based approach could help to find the most suitable conversion kit for the application, it is too costly for employment as a quality control step for individual samples.

Despite the possibility to use qBiCo for EC-converted DNA samples, which is currently detected at the same level as BC, its application is technically limited as it was designed and initially optimized for BC. For EC, qBiCo is only able to determine the conversion efficiency of the second enzymatic step: APOBEC conversion. This is the step in which all non-blocked cytosines are converted to thymines. The efficiency of the first step, blocking the 5mC nucleotides, cannot be measured by qBiCo, as the methylation state of CpGs cannot be assumed. At an incomplete 5mC blocking step, this could result in an under-representation of the methylation levels as unoxidated 5mC will be deaminated to DHU, while there would be no indication from the conversion efficiency index of qBiCo. However, recently a single-enzyme enzymatic conversion method is developed by NEB, for which this issue is not applicable [45]. To currently assess both enzymatic steps, it would be possible to design a qPCR assay similar to qBiCo, which targets a synthetic internal control with known methylation states.

With the emergence of many novel and automated [46] methylation analysis methods, future research could focus on extending the comparative quality control analysis between the current gold standards BC and EC shown here to include the most recent methods. Interestingly, method development for DNA methylation analysis is currently at an unprecedented level, especially when combined with SNP calling in integrated genetic-epigenetic approaches [33, 34]. Although these methods differ from standard bisulfite sequencing, they all include some form of bisulfite- or enzymatic-based conversion, stressing the need for a cost-effective QC tool and comparative analysis of these conversions. Similarly to what we performed here, qBiCo can be employed to evaluate and validate various other commercially available conversion kits, which is particularly important for offering standardized experimental pipelines for large-scale epigenomic analysis.

## Conclusion

Our findings indicate that both DNA conversion methods have different strengths and weaknesses, at least for the genomic DNA inputs tested. BC shows a high converted DNA recovery, whereas EC causes less converted DNA fragmentation, characteristic to BC. EC is, therefore, also more robust to input DNA that is fragmented, such as cell-free DNA. Overall, our study provides the first independent benchmarking of bisulfite and enzymatic conversion. This study highlights the need for further improving conversion approaches at low DNA quality and quantity.

## Methods

### Whole blood samples

In total, 25 whole blood samples were obtained from healthy donors approved by the Medical Ethics Review Committee of the Erasmus MC University Medical Center Rotterdam. The blood samples were drawn in 10 ml Blood Collection tube, BD Vacutainer®  with K2EDTA additive (Becton Dickinson). These samples were stored at 4 $^\circ \hbox {C}$ for four weeks before being processed.

### Genomic DNA extraction & quantification

Genomic DNA (gDNA) was extracted from two times 200 $\upmu$l of whole blood using the QiaAmp DNA Mini kit (QIAGEN, Germany) following the “DNA purification from blood or body fluids (spin protocol)” according to the manufacturer’s instructions. gDNA was eluted in 200 $\upmu$l, while a double elution step was performed by aspirating the eluate and reapplying the solution to the top of the column to maximize the DNA yield. Extracted DNA was quantified using the $\rm{Quantifiler}^{\rm{TM}}$ Human DNA Quantification Kit (Applied Biosystems, Massachusetts, USA) in half-volume reactions. We did not choose for a multi-target quantification kit (Quantifiler Trio) to simultaneously assess degradation levels of the gDNA sample, because pilot results were not sufficiently robust on our qPCR instrument. Additionally, differences in assay design such as target and primer sequences would have made the evaluation of degradation levels pre- and post-conversion not directly comparable. Three blood gDNA samples were used for all experiments as part of the developmental validation of both conversion methods, while the remaining 22 samples were used for the assessment of low-quantity DNA samples at 10 ng conversions.

### Validation sample preparation

Various parameters were used for this assessment according to the SWGDAM (Scientific Working Group on DNA Analysis methods) validation [47] and MIQE (Minimum Information for Publication of Quantitative Real-Time PCR Experiments) guidelines [48]. Supplementary table 5 in additional file 1 summarizes the design of our validation study. Briefly, to test the repeatability, reproducibility and sensitivity of conversion, different gDNA amounts (1-100 ng) were compared either within one (repeatability) or two batches (reproducibility/sensitivity). To prepare these, each gDNA sample was serially diluted to a concentration of 20, 4, 2, 1 and 0.2 ng/$\upmu$l in 5 $\upmu$l aliquots, which were stored at -20°C until thawed for conversion.

Additionally, conversion method-specific adjustments to the experimental protocol were made to assess possible effects on the conversion parameters. First, protocol-dependent incubation times during conversion were adjusted. For BC, the 16 h-long sodium bisulfite incubation step was adjusted to either 12 or 20 h. For EC, both incubation steps were elongated: TET incubation from 1 h to 1.5 h and APOBEC incubation from 3 to 4 h. Additional 1- and 5-minute binding times in addition to the standard 3-minute protocol, were performed for both column- (BC) and bead-based (EC) elution. For BC, a double elution step during column purification was also performed.

Additionally, to assess the influence of methylation status on the conversion performance, artificial DNA mixtures with various global methylation percentages (0, 25, 50, 75 and 100%) were prepared by mixing corresponding volumes of a highly methylated gDNA standard (EpigenDx, Massachusetts, USA) and a low methylated gDNA standard (Epigendx), to a total of 100 ng. Solutions were mixed to obtain methylation percentages of 0, 25, 50, 75 and 100%.

Moreover, to test the robustness of both conversion methods, degraded DNA samples were simulated both by exposure to UV light (30, 60 and 120 s) using the Bio-Link BLC-E254 UV irradiation system (Vilber, France) and sonication prior to conversion. For the latter, gDNA samples were sheared to sizes of 150, 500 and 1000 bp by Covaris S220 (Covaris, Massachusetts, USA) with the E220 intensifier in 55 $\upmu$l AFA Fiber Snap-Cap microTUBES (Covaris) as recommended by the supplier. DNA fragment size was confirmed by 1% agarose gel electrophoresis (Supplementary Figure 4).

The presence of common extracted DNA contaminants hematin and proteinase were also analyzed for their effects on the conversion performance [49]. Particularly, three levels of hematin were added to the extracted gDNA samples to final concentrations of 0, 100 and 200 $\upmu$M. Prior to addition to the gDNA sample hematin porcine (Sigma Aldrich, Missouri, USA) was dissolved in 1 M NaOH. The 0 $\upmu$M solution served as a negative control for the addition of the 1 M NaOH solution. On the other hand, three amounts of proteinase K (Qiagen) were added to the gDNA samples prior to conversion: 0.05, 0.1 and 0.2 mAU.

Finally, the stability of converted gDNA was tested both by measuring the effect of the storage time (four weeks at -20 °C) and the amount of freeze-thawing of the samples (no, five or ten freeze-thaw cycles). For each cycle, the sample was moved to -20 °C for 30 min and back to 20 °C for 30 times.

### DNA conversion

The conversion methods employed in this study differ in various ways. A reference amount of 100 ng gDNA was used if not otherwise specified and elution was performed in a volume of 20 $\upmu$l for all conversion reactions. To test all aforementioned parameters, a total of 142 bisulfite conversions and 139 enzymatic conversions were performed.

For bisulfite conversion, the EZ DNA methylation kit (Zymo Research, California, USA) was used, as it seems to be among the best performing commercially available kits [18]. All conversion steps were performed manually according to the manufacturer’s instructions. Genomic DNA samples were filled to a volume of 45 $\upmu$l before conversion, as required, and converted DNA samples were eluted in 20 $\upmu$l.

For enzymatic conversion, the NEBNext^®^ Enzymatic Methyl-seq Conversion Module (New England Biolabs, Massachusetts, USA) was used, as it is currently the only enzymatic conversion kit available in the market. All steps including bead cleanup were performed manually, but with adjustments to the protocol. Genomic DNA samples were filled to a volume of 28 $\upmu$l before conversion as required, but no fragmentation was performed, so that we do not introduce bias and solely obtain the fragmentation induced by the conversion protocol itself. Instead, as recommended by NEB, the fragmentation step was replaced with a DNA denaturation step at 90 °C for 10 min in a final concentration of 0.2x formamide. We were advised that there was no need for fragmentation of the gDNA with the adjusted formamide denaturation. We confirmed this via a trial experiment where we converted and compared between two fragmented and two non-fragmented samples (100 ng). We showed no negative effects on the conversion performance as tested by qBiCo (Additional file 1, Supplementary Table 6). For this trial, genomic DNA was sheared to a size of 300 bp by Covaris S220 (Covaris, Massachusetts, USA) with the E220 intensifier in 55 $\upmu$l AFA Fiber Snap-Cap microTUBES (Covaris) as recommended by the supplier. Elution of converted DNA was always performed in 20 $\upmu$l.

### qBiCo

Our previously published QC tool qBiCo was used to assess the performance of BC and EC conversion methods. qBiCo is a 5-plex qPCR assay targeting single-copy genes and repetitive elements of the human DNA in order to assess BC performance [18]. The five assays include the amplification of the Genomic, Converted, Long, Short and internal positive control (IPC) targets to calculate three performance indices: conversion efficiency, converted DNA concentration and converted DNA fragmentation. Two primer sets targeted the genomic and the converted version of the multi-copy human L1 repetitive element (LINE-1), respectively, providing the conversion efficiency. The long assay targeted the converted version of the single-copy gene (*TPT1*), whereas the short assay targeted the converted version of the single-copy gene *hTERT*. The combination of both assays was combined to determine the level of fragmentation of converted DNA, whereas the short assay alone was used for quantification of converted DNA. Finally, a set of primers was used to detect the IPC, which is added in the qPCR master mix to detect PCR inhibition. To quantify the copy numbers of each assay and eventually the qBiCo indices, an artificial DNA standard was created. More specifically, commercially synthesized DNA fragments for each of the four qBiCo assays (Converted, Long, Short, Genomic) were first produced as gBlock Gene Fragments by Integrative DNA Technologies (IDT, Iowa, USA). Then, the synthetic DNA fragments were mixed in known concentrations and ratios as previously tested and optimized (qBiCo-v2 [18]). The exact composition of the qBiCo standard in terms of copy number of each synthetic DNA fragment are also presented in Additional file 1, Supplementary table 4. To create the qBiCo standard curve, a serial dilution of the synthetic DNA standard was performed; specifically, a total of five standards were created by a 3-fold serial dilution in TE buffer.

For this study, we ran the qBiCo-v2 protocol as previously reported [18] on the Bio-rad CFX96 $rm{Touch}^{\rm{TM}}$ Real-Time PCR Detection system (Bio-Rad Laboratories, California, USA). Briefly, the qBiCo master mix consisted of: 5 $\upmu$l of 2x EpiTect Methylight PCR reagent (Qiagen), 1 $\upmu$l primer-probe mix, 1 $\upmu$l 25 mM MgCl_2_ (Thermo Fisher Scientific, Massachusetts, USA), 0.2 $\upmu$l 20 mg/ml BSA (New England Biolabs), 1.3 $\upmu$l nuclease-free water (Thermo Fisher Scientific), 0.5 $\upmu$l IPC (6000 copies/$\upmu$l) in TE buffer (Thermo Fisher Scientific) and 1 $\upmu$l of the to-be-analyzed sample. Detection thresholds were set empirically and according to standard qPCR practices. The qBiCo standard curves and their corresponding qPCR parameters for each of the runs can be found in Additional file 1.

### Data analysis

qPCR data was exported through the CFX Maestro software version 2.2 (Bio-Rad Laboratories). Raw qPCR data was pre-processed through the qBiCo processing R script (Additional file 3) obtaining the standard curves per conversion experiment (Additional file 1) and the samples Cq values (Additional file 1). Cq values were then converted into qBiCo indices: conversion efficiency (Eq. 1), converted DNA concentration and fragmentation (Eq. 3). With the known input amount for both conversion methods, the converted DNA recovery index was calculated directly from the DNA concentration (Eq. 2). Below we present all formulas used to calculate the qBiCo indexes:

1 $$\begin{aligned} & \text {BC\,efficiency}\, {(\%)} = \frac{[\text{LINE1}\, \text{Converted}]}{[\text{LINE1}\, \text{Converted}] + [\text{LINE1}\, \text{Genomic}]} \times 100 \% \end{aligned}$$

2 $$\begin{aligned} & {\text{BC}\, \text{DNA}\, \text{Recovery}} = \frac{[\textit{hTERT}\, \text{Short}]}{\text{Total} \ \text{input}} \end{aligned}$$

3 $$\begin{aligned} & \text {BC \,DNA \,fragmentation \,index} = \frac{[\textit{hTERT}\, \text{Short}]}{[\textit{TPT1}\, \text{Long}]} \end{aligned}$$

All qBiCo index results can be found in Additional file 1. Plotting and data analysis was performed through the Data analysis R script (Additional file 4). Finally, statistical tests were performed on RStudio version 1.2.5001, while plots were created with the ggplot2 package [50]. Specifically, statistical significance was assessed only for the samples within the repeatability, reproducibility & sensitivity parameter due to sufficient sample size. Pairwise unpaired Wilcoxon Rank Sum tests were performed for sample groups between conversion methods grouped per DNA input amount by the compare_means function in the ggpubr library. Comparing both conversion methods was performed by ANCOVA with the anova_test function from the rstatix library. More information can be found in Additional file 1.

## Supplementary Information

Supplementary file 1. PDF file containing qBiCo standard curves and corresponding parameters. Supplementary Figure 1. qBiCo standard curves per BC run; Supplementary Figure 2. qBiCo standard curves per EC run; Supplementary Figure 3. qBiCo standard curves for BC and EC conversion of 22 10 ng gDNA samples; Supplementary Figure 4. DNA degradation assessment of sonicated samples; Supplementary Table 1. qBiCo standard curve parameters per BC conversion experiment. (Efficiency, R squared value of linear regression fit, slope and intercept); Supplementary Table 2. qBiCo standard curve parameters per EC conversion experiment. (Efficiency, R squared value of linear regression fit, slope and intercept); Supplementary Table 3. qBiCo standard curve parameters for BC and EC conversion of 22 10 ng gDNA samples. (Efficiency, R squared value of linear regression fit, slope and intercept); Supplementary Table 4. qBiCo gblock concentration (copy numbers/microliter) per run; Supplementary Table 5. Study design; Supplementary Table 6. Effect of fragmentation on enzymatic conversion performance measured by qBiCo.

Supplementary file 2. R file containing the script to compute the qBiCo indices from qPCR data.

Supplementary file 3. qBiCo index data tab contains the qBiCo results of each converted sample in duplicate per row. Columns contain the following labels: Sample, Well, Conversion efficiency, DNA concentration, Fragmentation, Experiment, DNA input, Elution volume, Expected concentration, Conversion method and Recovery. Conversion experiment labeled tabs (BC1, EC1, etc.) include the Cq values of each converted sample in duplicate. Columns contain the following labels: Well, Fluor, Target, Sample, Cq, DNA input, Elution volume, Excluded (1 if excluded based on manual curation of qPCR data). The graphs sample list tab shows which samples are used for analysis of each validation parameter. The statistics comparative tests tab contains the results of the comparative statistical tests. The statistics pairwise tests tab contains the results of the pairwise statistical tests.

Supplementary file 4. R file containing the script to perform statistical tests and create figures.

## Data Availability

All data generated for this study as well as the employed scripts for analysis are included in the supplementary files of this article.

## Abbreviations

5mc: Methylated cytosine
BC: Bisulfite conversion
CpG: Cytosine guanine dinucleotide
DHU: Dihydrouracil
EC: Enzymatic conversion
FT cyles: Freeze-thaw cycles
qBiCo: Bisulfite converted quantification and qualification assay
TC: Tet-assisted pyridine borane conversion
