# Supplementary material for: Comparative performance evaluation of bisulfite- and enzyme-based DNA conversion methods
Source: Clin Epigenetics. 2025 Apr 3;17:56. doi: 10.1186/s13148-025-01855-7 (PMC11969950; doi:10.1186/s13148-025-01855-7)
Supplement: Supplementary file 1 — PDF file containing qBiCo standard curves and corresponding parameters. Supplementary Figure 1. qBiCo standard curves per BC run; Supplementary Figure 2. qBiCo standard curves per EC run; Supplementary Figure 3. qBiCo standard curves for BC and EC conversion of 22 10 ng gDNA samples; Supplementary Figure 4. DNA degradation assessment of sonicated samples; Supplementary Table 1. qBiCo standard curve parameters per BC conversion experiment. (Efficiency, R squared value of linear regression fit, slope and intercept); Supplementary Table 2. qBiCo standard curve parameters per EC conversion experiment. (Efficiency, R squared value of linear regression fit, slope and intercept); Supplementary Table 3. qBiCo standard curve parameters for BC and EC conversion of 22 10 ng gDNA samples. (Efficiency, R squared value of linear regression fit, slope and intercept); Supplementary Table 4. qBiCo gblock concentration (copy numbers/microliter) per run; Supplementary Table 5. Study design; Supplementary Table 6. Effect of fragmentation on enzymatic conversion performance measured by qBiCo. [file 13148_2025_1855_MOESM1_ESM.pdf]

# Additional file 1 – qBiCo standard curves and study design

The qBiCo standard curve was created by using a DNA mixture containing synthetic DNA fragments as targets for each of the four assays. The copy number of each synthetic DNA fragment in the first standard of each qBiCo run can be seen in supplementary table 4. The four consecutive standards were created by 3-fold serial dilution in TE buffer. Supplementary Figure 1 and 2 show the standard curves of each separate conversion experiment for the bisulfite and enzymatic conversions of 100 ng gDNA samples. Supplementary Figure 3 shows the qBiCo standard curves for the set of 22 10 ng samples. The summary statistics of each qBiCo assay fit can be seen in Supplementary Table 1, Supplementary Table 2 and Supplementary Table 3. For an overview of the study design, indicating which samples were converted for which developmental validation parameter see Supplementary Table 5.

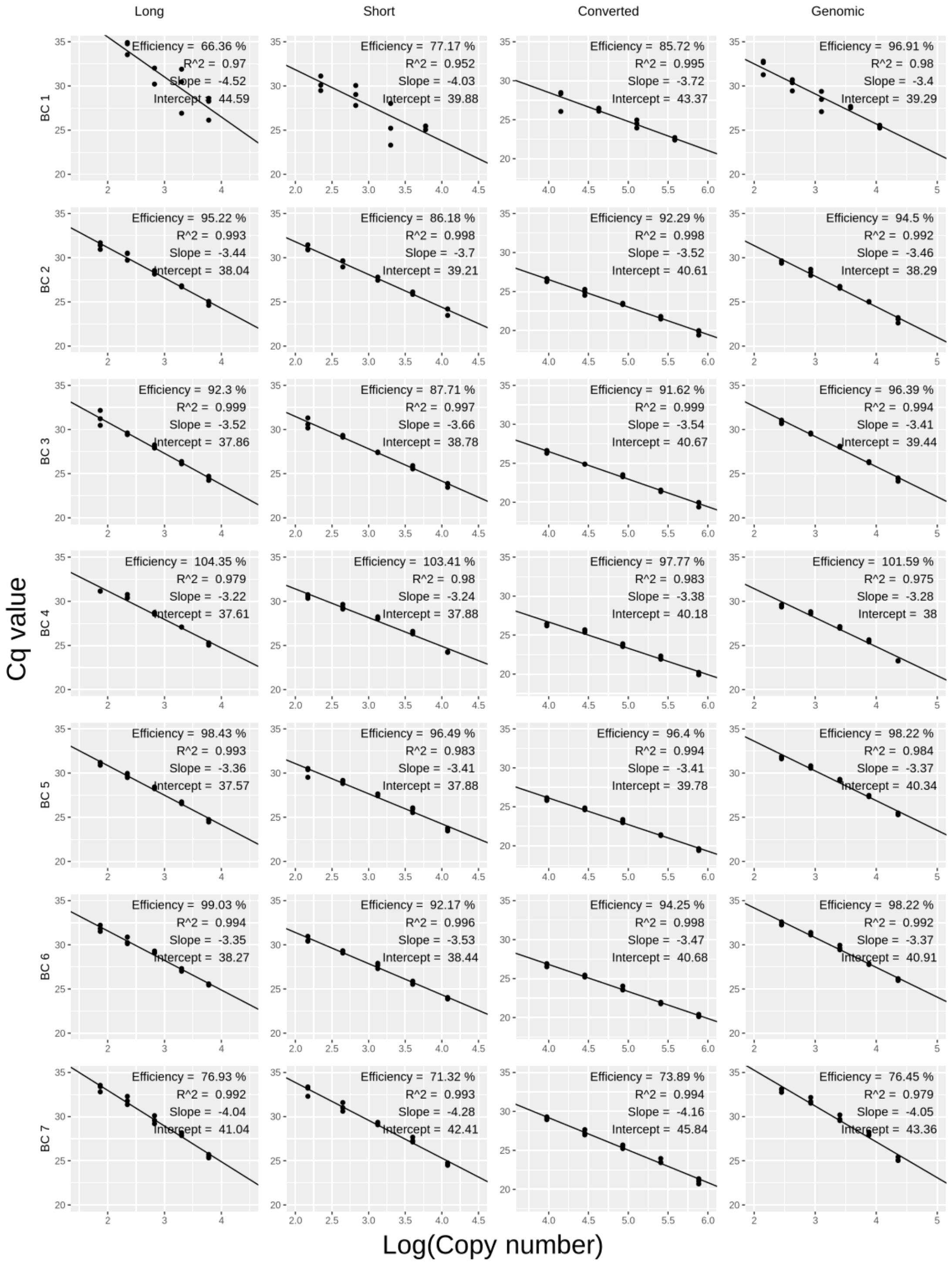


*Supplementary Figure 1. qBiCo standard curves of the four assays per BC conversion experiment. Each column indicates the qBiCo assay and each row indicates a conversion experiment.*

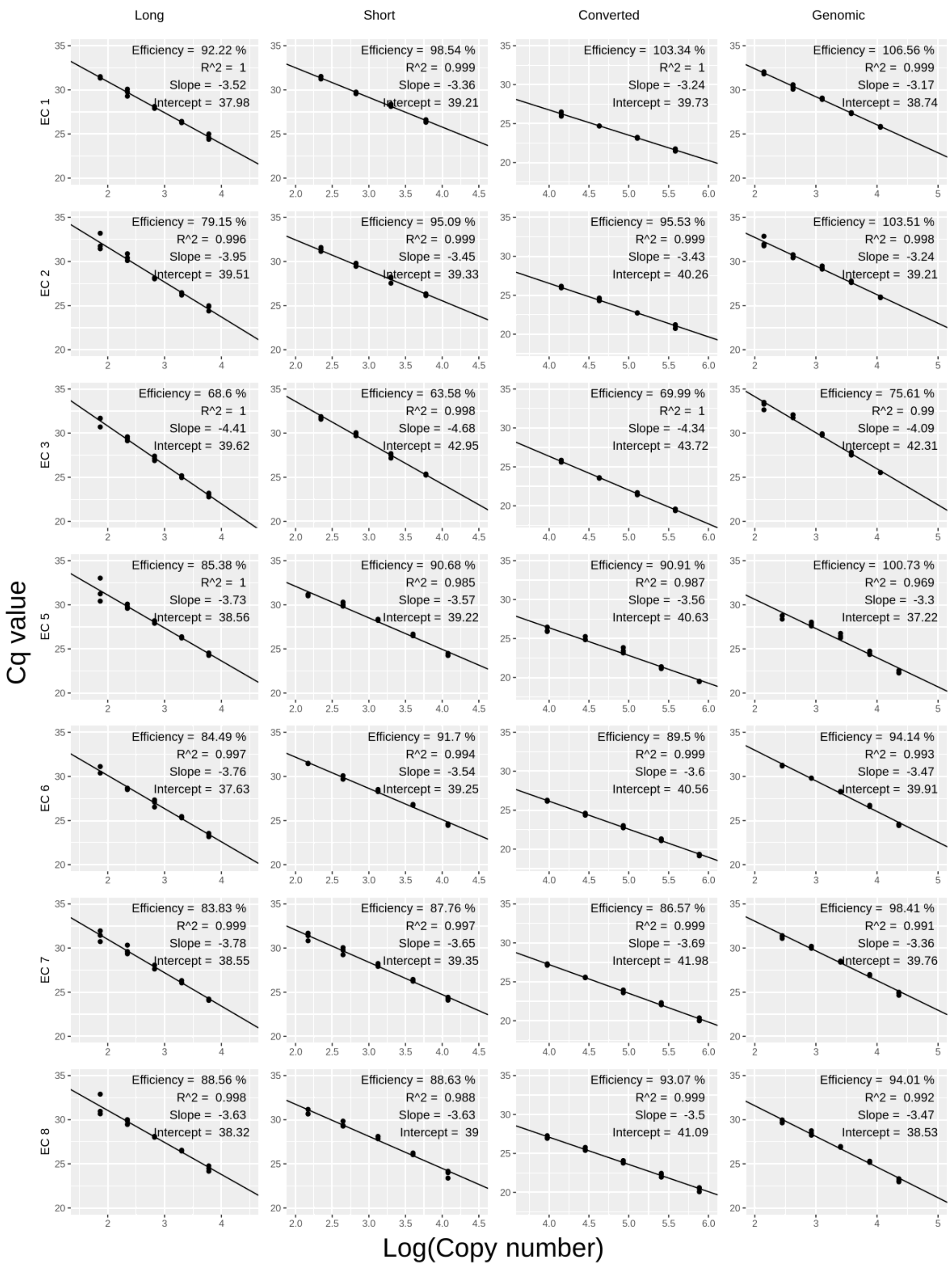


*Supplementary Figure 2. qBiCo standard curves of the four assays per EC conversion experiment. Each column indicates the qBiCo assay and each row indicates a conversion experiment.*

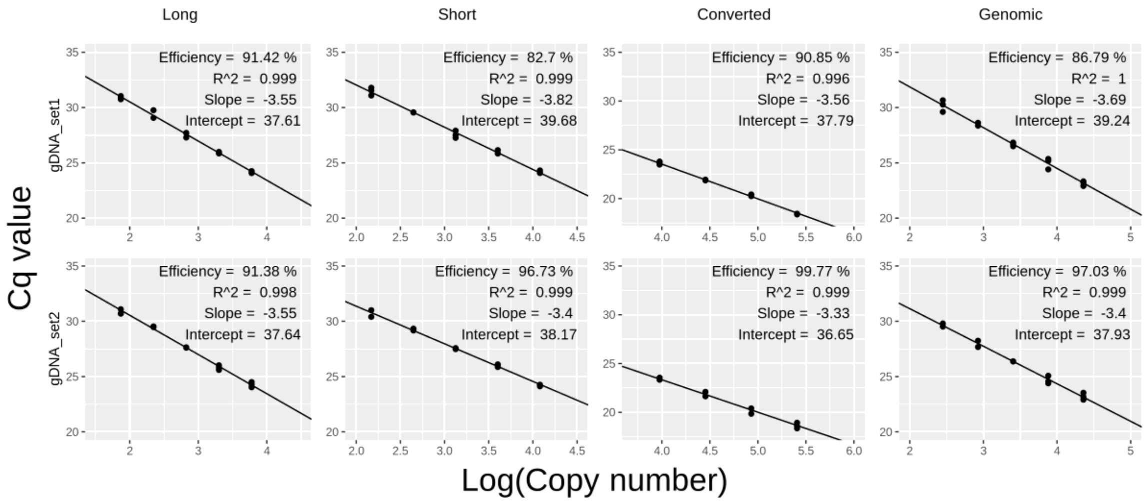


*Supplementary Figure 3. qBiCo standard curves of the four assays per qBiCo assay for the BC and EC conversion of 22 10 ng gDNA samples. Each column indicates the qBiCo assay and each row indicates a conversion experiment.*

## Sonication assessment

To test the robustness of the conversion methods, conversions with degraded DNA input were performed. Genomic DNA samples were sheared to ~150 bp, ~500 bp and ~1000 bp by sonication with the Covaris S220 instrument and E220 intensifier in 55 µl AFA Fiber Snap-Cap microTUBES (Covaris). To assess this degradation, the fragment lengths were confirmed by 1 \% agarose gel electrophoresis (Supplementary Figure 4). The incremental fragmentation of all three sonication levels can be seen. The obtained fragment length ranges are as expected.

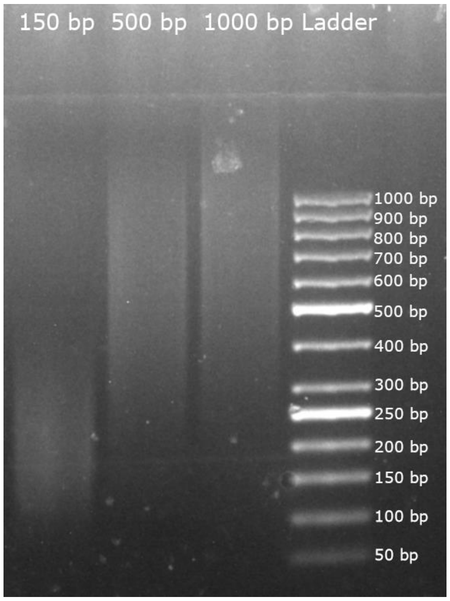


*Supplementary Figure 4. Degradation assessment of sonicated samples on a 1% agarose gel. Genomic DNA samples were sheared to ~150 bp, ~500 bp and ~1000 bp by sonication*

*Supplementary Table 1. qBiCo standard curve parameters of the four assays per BC conversion experiment.*

| Experiment | Assay | Fluorophore | PCR efficiency (%) | r_squared | intercept | slope |
|---|---|---|---|---|---|---|
| BC_1 | Long | FAM | 66.4 | 0.970 | 44.6 | -4.52 |
| | Short | HEX | 77.2 | 0.952 | 39.9 | -4.03 |
| | Converted | TEX615 | 85.7 | 0.995 | 43.4 | -3.72 |
| | Genomic | Cy5 | 96.9 | 0.980 | 39.3 | -3.40 |
| BC_2 | Long | FAM | 95.2 | 0.993 | 38.0 | -3.44 |
| | Short | HEX | 86.2 | 0.998 | 39.2 | -3.70 |
| | Converted | TEX615 | 92.3 | 0.998 | 40.6 | -3.52 |
| | Genomic | Cy5 | 94.5 | 0.992 | 38.3 | -3.46 |
| BC_3 | Long | FAM | 92.3 | 0.999 | 37.9 | -3.52 |
| | Short | HEX | 87.7 | 0.997 | 38.8 | -3.66 |
| | Converted | TEX615 | 91.6 | 0.999 | 40.7 | -3.54 |
| | Genomic | Cy5 | 96.4 | 0.994 | 39.4 | -3.41 |
| BC_4 | Long | FAM | 104.3 | 0.979 | 37.6 | -3.22 |
| | Short | HEX | 103.4 | 0.980 | 37.9 | -3.24 |
| | Converted | TEX615 | 97.8 | 0.983 | 40.2 | -3.38 |
| | Genomic | Cy5 | 101.6 | 0.975 | 38.0 | -3.28 |
| BC_5 | Long | FAM | 98.4 | 0.993 | 37.6 | -3.36 |
| | Short | HEX | 96.5 | 0.983 | 37.9 | -3.41 |
| | Converted | TEX615 | 96.4 | 0.994 | 39.8 | -3.41 |
| | Genomic | Cy5 | 98.2 | 0.984 | 40.3 | -3.37 |
| BC_6 | Long | FAM | 99.0 | 0.994 | 38.3 | -3.35 |
| | Short | HEX | 92.2 | 0.996 | 38.4 | -3.53 |
| | Converted | TEX615 | 94.2 | 0.998 | 40.7 | -3.47 |
| | Genomic | Cy5 | 98.2 | 0.992 | 40.9 | -3.37 |
| BC_7 | Long | FAM | 76.9 | 0.992 | 41.0 | -4.04 |
| | Short | HEX | 71.3 | 0.993 | 42.4 | -4.28 |
| | Converted | TEX615 | 73.9 | 0.994 | 45.8 | -4.16 |
| | Genomic | Cy5 | 76.5 | 0.979 | 43.4 | -4.05 |

*Supplementary Table 2. qBiCo standard curve parameters of the four assays per EC conversion experiment.*

| Experiment | Assay | Fluorophore | PCR efficiency (%) | r_squared | intercept | slope |
|---|---|---|---|---|---|---|
| EC_1 | Long | FAM | 92.2 | 1.000 | 38.0 | -3.52 |
| | Short | HEX | 98.5 | 0.999 | 39.2 | -3.36 |
| | Converted | TEX615 | 103.3 | 1.000 | 39.7 | -3.24 |
| | Genomic | Cy5 | 106.6 | 0.999 | 38.7 | -3.17 |
| EC_2 | Long | FAM | 79.2 | 0.996 | 39.5 | -3.95 |
| | Short | HEX | 95.1 | 0.999 | 39.3 | -3.45 |
| | Converted | TEX615 | 95.5 | 0.999 | 40.3 | -3.43 |
| | Genomic | Cy5 | 103.5 | 0.998 | 39.2 | -3.24 |
| EC_3 | Long | FAM | 68.6 | 1.000 | 39.6 | -4.41 |
| | Short | HEX | 63.6 | 0.998 | 42.9 | -4.68 |
| | Converted | TEX615 | 70.0 | 1.000 | 43.7 | -4.34 |
| | Genomic | Cy5 | 75.6 | 0.990 | 42.3 | -4.09 |
| EC_5 | Long | FAM | 85.4 | 1.000 | 38.6 | -3.73 |
| | Short | HEX | 90.7 | 0.985 | 39.2 | -3.57 |
| | Converted | TEX615 | 90.9 | 0.987 | 40.6 | -3.56 |
| | Genomic | Cy5 | 100.7 | 0.969 | 37.2 | -3.30 |
| EC_6 | Long | FAM | 84.5 | 0.997 | 37.6 | -3.76 |
| | Short | HEX | 91.7 | 0.994 | 39.3 | -3.54 |
| | Converted | TEX615 | 89.5 | 0.999 | 40.6 | -3.60 |
| | Genomic | Cy5 | 94.1 | 0.993 | 39.9 | -3.47 |
| EC_7 | Long | FAM | 83.8 | 0.999 | 38.6 | -3.78 |
| | Short | HEX | 87.8 | 0.997 | 39.4 | -3.65 |
| | Converted | TEX615 | 86.6 | 0.999 | 42.0 | -3.69 |
| | Genomic | Cy5 | 98.4 | 0.991 | 39.8 | -3.36 |
| EC_8 | Long | FAM | 88.6 | 0.998 | 38.3 | -3.63 |
| | Short | HEX | 88.6 | 0.988 | 39.0 | -3.63 |
| | Converted | TEX615 | 93.1 | 0.999 | 41.1 | -3.50 |
| | Genomic | Cy5 | 94.0 | 0.992 | 38.5 | -3.47 |

*Supplementary Table 3. qBiCo standard curve parameters of the four assays per qBiCo assay for the BC and EC conversion of 22 10 ng gDNA samples.*

| Experiment | Assay | Fluorophore | PCR efficiency (%) | r_squared | intercept | slope |
|---|---|---|---|---|---|---|
| Set_1 | Long | FAM | 91.4 | 0.999 | 37.6 | -3.55 |
| | Short | HEX | 82.7 | 0.999 | 39.7 | -3.82 |
| | Converted | TEX615 | 90.8 | 0.996 | 37.8 | -3.56 |
| | Genomic | Cy5 | 86.8 | 1.000 | 39.2 | -3.69 |
| Set_2 | Long | FAM | 91.4 | 0.998 | 37.6 | -3.55 |
| | Short | HEX | 96.7 | 0.999 | 38.2 | -3.40 |
| | Converted | TEX615 | 99.8 | 0.999 | 36.6 | -3.33 |
| | Genomic | Cy5 | 97.0 | 0.999 | 37.9 | -3.40 |

*Supplementary Table 4. qBiCo gBlock amount per assay for each conversion experiment.*

| Experiment | Copies_Long | Copies_Short | Copies_Converted | Copies_Genomic |
|---|---|---|---|---|
| BC_1 | 6000 | 6000 | 384000 | 11400 |
| BC_2 | 6000 | 12000 | 768000 | 22800 |
| BC_3 | 6000 | 12000 | 768000 | 22800 |
| BC_4 | 6000 | 12000 | 768000 | 22800 |
| BC_5 | 6000 | 12000 | 768000 | 22800 |
| BC_6 | 6000 | 12000 | 768000 | 22800 |
| BC_7 | 6000 | 12000 | 768000 | 22800 |
| EC_1 | 6000 | 6000 | 384000 | 11400 |
| EC_2 | 6000 | 6000 | 384000 | 11400 |
| EC_3 | 6000 | 6000 | 384000 | 11400 |
| EC_5 | 6000 | 12000 | 768000 | 22800 |
| EC_6 | 6000 | 12000 | 768000 | 22800 |
| EC_7 | 6000 | 12000 | 768000 | 22800 |
| EC_8 | 6000 | 12000 | 768000 | 22800 |
| Set_1 | 6000 | 12000 | 768000 | 22800 |
| Set_2 | 6000 | 12000 | 768000 | 22800 |

*Supplementary table 5. Study design overview. Conversions performed per developmental validation parameter. Three gDNA samples were used in this study.*

| Parameter | Description | Conversions per gDNA sample |
|---|---|---|
| Repeatability | DNA amount (100,20,10,5,1 ng) | 3 for 100 ng and lowest repeatable amount (BC: 5 ng, EC: 10 ng); 2 for other amounts |
| Reproducibility and Sensitivity | DNA amount (100,20,10,5,1 ng) | 5 for 100 ng and lowest repeatable amount (BC: 5 ng, EC: 10 ng); 2 for other amounts |
| Recovery: Elution | 1 and 5 minutes binding time, double elution (BC only) | 1 |
| Incubation time | BC (12 and 20 hours); EC (TET: 1.5 and APOBEC 4 hours) | 1 |
| Artificial methylation | 100, 75, 50, 25 and 0 % methylation | 2 (artificial DNA) |
| Robustness: UV | 0, 30, 60 and 120 seconds UV | 1 |
| Robustness: Sonication | 150, 500, 1000 bp and non sonicated control | 1 |
| Inhibition: Hematin | 0, 100 and 200 µM and solute control | 1 |
| Inhibition: Proteinase | 0, 0.05, 0.1 and 0.2 mAU | 1 |
| Stability: Storage | 0 and 4 weeks | 1 |
| Stability: Freeze-thaw cycles | 0, 5 and 10 FT-cycles | 1 |

**Enzymatic conversion trial**

The effect of shearing the gDNA to 300 bp by sonication prior to enzymatic conversion was tested. With the denaturation adjustment to the enzymatic conversion protocol, it was possible to perform enzymatic conversion without fragmentation. Recovery and conversion efficiency were higher for the samples which were not fragmented. As expected the fragmented samples showed a higher fragmentation level than the non-fragmented samples.

*Supplementary Table 6. Effect of fragmentation on enzymatic conversion performance measured by qBiCo.*

| Duplicate | Fragmentation | Input (ng) | Output (ng) | Recovery | Conversion efficiency | Fragmentation |
|---|---|---|---|---|---|---|
| 1 | - | 106.4 | 69.8 | 66% | 99.1% | 2.19 |
| 2 | - | 106.4 | 56.7 | 53% | 99.1% | 2.16 |
| 1 | 300 bp Covaris | 83.2 | 47.2 | 57% | 97.8% | 5.14 |
| 2 | 300 bp Covaris | 83.2 | 38.9 | 47% | 98.4% | 6.23 |
